# Supplementary material for: Associations of Autistic Traits and Autism with Incontinence and Constipation in a UK Birth Cohort
Source: J Autism Dev Disord. 2024 Dec 7;56(5):1999–2008. doi: 10.1007/s10803-024-06663-1 (PMC13190406; doi:10.1007/s10803-024-06663-1)
Supplement: Supplementary file 1 — Supplementary file1 (DOCX 78 kb) [file 10803_2024_6663_MOESM1_ESM.docx]

# **Supplementary Materials**

**Supplementary Text 1**

The sociability subscale of the Emotionality Activity and Sociability (EAS) temperament measure is used to assess an individual's preference for being with others rather than being alone. It measures the extent to which an individual seeks social interaction, shares activities, and desires attention from others (Bould et al., 2013). The sociability subscale is one of the four subscales of the EAS measure, which also includes emotionality, activity, and impulsivity. The EAS measure is widely used to assess temperament in children and has been found to have good stability over time (Bould et al., 2013). The items are evaluated using a five-point Likert scale, which ranges from one (not typical) to five (very typical). The summation of the items within the scale is divided by five to derive the corresponding score. The items in the sociability subscale include.

1: Likes to be with people.

2: Prefers playing with others rather than alone.

3: Finds people more stimulating than anything else.

4: Is something of a loner.

5: When alone, the child feels isolated.

**Supplementary Text 2**

The Repetitive Behaviour Scale is a tool used to assess and describe repetitive and restricted behaviours in various psychiatric syndromes, including autism spectrum disorders ([Bourreau et al., 2009](#_ENREF_3)). It provides a standardized and accurate description of these behaviours, allowing for a better understanding of their clinical dimension ([Bourreau et al., 2009](#_ENREF_3)). The scale has been found to have good psychometric qualities, including good interrater reliability, internal consistency, and content validity ([Bourreau et al., 2009](#_ENREF_3)). The scale has potential clinical applications and can be used in research, treatment, and clinical practice. In the ALSPAC, this scale was developed from the answers to four questions in the questionnaire sent to the mother at 69 months and these were as follows.

(a) How often does he/she repeatedly rock his head or body for no reason?

(b) How often does he/she have a tic or twitch?

(c) How often does he/she have other unusual behaviour?

(d) How often does he/she stumble or get stuck on words, or repeat them many times? (example, I I I I want a sweet)? The responses to each question were coded as: “often/always =3”, “sometimes =2”, and “never =1”.

**Supplementary Text 3**

The Social and Communication Disorders Checklist, also known as SCDC, is a twelve-item checklist that was designed to allow for quick and efficient evaluation of autistic traits. Within this set of questions, nine are directed towards measuring abnormalities in the aspects of the autistic triad that pertain to reciprocal social interaction and communication skills ([Skuse et al., 2005](#_ENREF_35)). Each item on the scale is evaluated based on its occurrence within the past six months and whether the associated statements are “not true”, “quite or sometimes true" or "very or often true." The assigned scores are “0”, “1”, or “2”, which culminate in a maximum score of twenty-four ([Skuse et al., 2005](#_ENREF_35)). The SCDC has strong internal consistency and test-retest stability ([Solmi et al., 2021](#_ENREF_36)). Items in the SCDC scale are as follows;
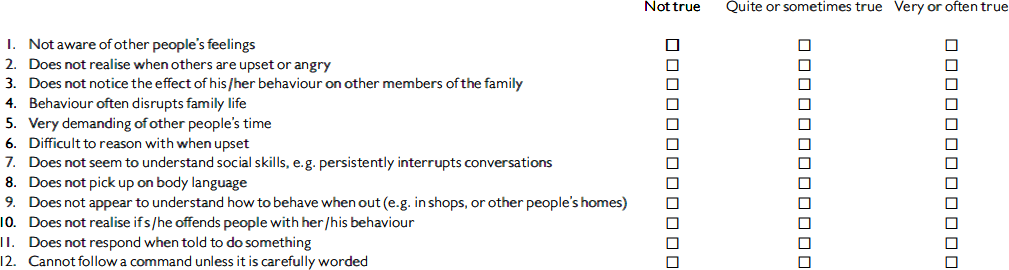


**Supplementary Text 4**

The children communication checklist (CCC) is a standardized questionnaire comprising seventy items, which has been specifically developed to evaluate the communicative abilities of children ([Lane et al., 2019](#_ENREF_24)). This instrument is highly effective in detecting significant communicative difficulties among children ([Lane et al., 2019](#_ENREF_24)). The CCC-2 questionnaire consists of ten subscales, each of which assesses a different aspect of communication, including (A) speech, (B) syntax, (C) semantics, (D) coherence, (E) inappropriate initiation, (F) stereotyped language, (G) use of context, (H) nonverbal communication, (I) social relations, and (J) interests. Each of these subscales is comprised of seven items, five of which are related to communicative difficulties, and two of which are related to communicative strengths ([Lane et al., 2019](#_ENREF_24)). Analyses of traits predictive of autism in ALSPAC showed that the coherence subscale of the CCC performed better than the other CCC scales ([Golding et al., 2017](#_ENREF_15)), and therefore that was used in our study. The coherence scale is made up of eight items which are as follows.

1. It is sometimes hard to make sense of what he is saying because it seems illogical or disconnected.

2. Conversation with him can be enjoyable and interesting.

3. Can give an easy-to-follow account of a past event such as a birthday party or holiday.

4. Can talk clearly about what he plans to do in the future (example, tomorrow, or next week)

5. Would have difficulty in explaining to a younger child how to play a simple game such as ‘‘snap’’.

6. Has difficulty in telling a story, or describing what he has done, in an orderly sequence of events.

7. Uses terms like ‘‘he’’ or ‘‘it’’ without making it clear what he is talking about.

8. Doesn’t seem to realise the need to explain what he is talking about to someone who doesn’t share his experiences; for instance, might talk about ‘‘Johnny’’ without explaining who he is.

The items included in the questionnaire are evaluated using a Likert scale, which provides an assessment of the frequency of communicative problems and strengths. The scale ranges from zero, representing less than once a week or never, to three, representing several times a day or always ([Lane et al., 2019](#_ENREF_24)).

**Supplementary Text 5**

The factor-mean-score as a composite measure of autistic traits was derived using principal components analysis (PCA) ([Steer et al., 2010](#_ENREF_38)). Ninety-three traits that potentially indexed the emergence and presence of autism were selected for factor analysis ([Steer et al., 2010](#_ENREF_38)). The parental questionnaire, as well as observational and standardized tests, were used to select measures that were included in the factor analysis ([Bolton et al., 2012](#_ENREF_2)). These measures were collected at various time points in development. The factor analysis identified seven principal factors which related to verbal ability, language acquisition, social understanding, semantic-pragmatic skills, repetitive stereotyped behaviour, articulation, and social inhibition ([Bolton et al., 2012](#_ENREF_2)). A single common autistic trait score was derived by taking the mean score of these seven factors and low scores on this derived variable reflected more autistic traits ([Bolton et al., 2012](#_ENREF_2)).

| **Supplementary table 1**. *Characteristics of the studies included in the literature review* | | | | | | | | | | |
| --- | --- | --- | --- | --- | --- | --- | --- | --- | --- | --- |
| **Authors (date)** | **Title** | **Objectives** | **Study design** | **Participants** | **Sample size** | **Exposures** | **Outcomes** | **Confounders adjusted for** | **Findings** | **Limitations** |
| Brittany Peters, Kent Williams, Phillip Gorrindo, Daniel Rosenberg, Evon Lee, Pat Levitt, Jeremy Veenstra-Vanderweele, Á Lee, and Á Veenstra-Vanderweele (2013) | Rigid–Compulsive behaviours are associated with mixed bowel symptoms in autism spectrum disorder. | The study evaluated the association between gastrointestinal symptoms and measures of rigid compulsive behaviours in children ages 2-17 years. | Cross-sectional study. | Children between the ages of 2-17 years with autism spectrum disorder. | Not indicated | Parental report of repetitive behaviour, parental report of compulsive behaviour, clinician diagnosis of obsessive-compulsive disorder, and report of rituals observed on the autism diagnostic observation schedule. | Constipation and co-occurring diarrhoea or underwear staining in children with autism spectrum disorder. | Age, sex, race/ethnicity, nonverbal IQ, presence of anxiety, depression, and attention-deficit/hyperactivity disorder (ADHD). | The findings of the study suggest that there is an association between rigid-compulsive behaviours and severe constipation and co-occurring diarrhoea or underwear staining in children with autism spectrum disorder. | The study was cross-sectional; hence, it cannot establish causality between rigid-compulsive behaviours and gastrointestinal symptoms because of the potential for reverse causality. |
| Justine Niemczyk, Roman Fischer, Catharina Wagner, Alina Burau, Theresa Link, and Alexander Von Gontard (2019). | Detailed assessment of incontinence, psychological problems and parental Stress in children with autism spectrum disorder. | The paper examined incontinence, psychological symptoms, parental stress, and psychopathology in children with autism spectrum disorders (ASD) compared to a control group of typically developing children. | Case-control study. | The study included 51 children (43 boys, mean age = 9.7 years) with autism spectrum disorder (ASD) and 53 matched controls (43 boys, mean age = 10.2 years). | The sample size for the study was 51 children with autism spectrum disorder and 53 matched controls. | Autism Spectrum Disorder (ASD) diagnosis. | Different types of incontinence, including enuresis (bedwetting), daytime urinary incontinence, and faecal incontinence**,** psychological symptoms**,** and parental stress. | The study did not adjust for confounders; however, the control subjects were matched on gender and sex. | The findings of this paper suggest that children with autism spectrum disorder (ASD) have a higher prevalence of enuresis (bedwetting) and daytime urinary incontinence (DUI) compared to typically developing children. | Small sample size and unadjustment for confounders. |
| M Equit, A Von Gontard, M Pirrung, J Niemczyk (2015). | Incontinence in children with autism spectrum disorder. | The objective of the paper was to assess the Prevalence of incontinence, lower urinary tract symptoms (LUTS), and psychological symptoms/disorders in children with Autism Spectrum Disorder (ASD) compared to controls. | Case-control study | The participants were 40 children with ASD with a mean age of 11.3 years and 43 age-matched control children with a mean age of 10.7 years. | 40 children with ASD and 43 age-matched control children. | Autism diagnosis. | Incontinence. | Sex and IQ were adjusted for in the analysis. | The study found that children with ASD had increased rates of nocturnal enuresis (30.0% vs 0%) and daytime urinary incontinence (25.0% vs 4.7%) compared to controls. Children with ASD also had delayed daytime bladder control (20.5% vs 0%) and bowel control (42.5% vs 7.5%) compared to controls. Children with ASD had a higher score for lower urinary tract symptoms (LUTS) | Small sample size and inadequate adjusted for confounders. |
| Marilena Gubbiotti, Sandro Elisei, Chiara Bedetti, Moreno Marchiafava, and Antonella Giannantoni (2019). | Urinary and bowel disfunction in autism spectrum disorder: a prospective, observational study. | To evaluate the prevalence and types of bladder and bowel dysfunction in young and adult patients affected by Autism ASD, and to investigate the potential contribution of pharmacological agents assumed by these patients to the development and maintenance of bladder and bowel dysfunction. | Prospective, observational study. | 27 adults and 20 children/teens with ASD, as well as a matched group of typically developing subjects. | 47 patients with ASD and matched group of typically developing subjects. | Autism diagnosis. | Bladder and bowel disfunctions. | The study did not adjust for confounders, but control subjects were matched on age and sex**.** | The study found that young and adult patients with ASD present with a high prevalence of incontinence. | The study was conducted at a single centre, which may limit the generalizability of the findings. The sample size was relatively small, which may limit the statistical power of the study and there was a very short follow-up period. |
| Holly A. Harris PhD Nadia Micali MD, PhD, Henriette A. Moll MD, PhD, Ina van Berckelaer-Onnes PhD 7 Manon Hillegers MD, PhD, and Pauline W. Jansen PhD (2021). | The role of food selectivity in the association between child autistic traits and constipation. | The study aimed to contribute to the understanding of the relationship between autistic traits, food selectivity, and constipation symptoms, and to inform potential behavioural interventions and support for families of children with autistic traits. | Population-based prospective cohort study. | The participants in the study were drawn from the Generation R birth cohort, a population-based sample from Rotterdam, the Netherlands | The total number of participants included in the study was 2,818 | Child autistic traits. | Constipation symptoms. | Child baseline constipation, BMI, IQ, and maternal education. | The study found a positive association between parent-reported child autistic traits and constipation symptoms (r = 0.08, p < .001). Food selectivity was identified as a significant mediator in the association between autistic traits and constipation symptoms (β = 0.008, 95% CI: 0.002, 0.014). | Omission of important confounders such as social class, maternal depression, and anxiety. The study relied on parent-reported measures for both autistic traits and constipation which could lead to misclassification. |
| Samar Ibrahim, Robert Voigt, Slavica Katusic, Amy Weaver, and William Barbaresi (2009). | Incidence of gastrointestinal symptoms in children: A Population-Based Study. | To determine whether children with ASD have an increased incidence of gastrointestinal (GI) symptoms compared to matched controls in a population-based sample. | The study was a population-based cohort study. | 124 children aged < 21 years resident in Olmsted County who fulfilled DSM-IV based criteria for a research diagnosis of autism and 2 matched controls. | The total sample size was 372 (124 cases and 248 controls). | ASD diagnosis. | GI symptoms including constipation. | The paper did not mention any confounders that were adjusted for in the study. However, the controls were matched on gender, age, year of first registration, and duration of follow-up. | The study found that children with autism had an increased incidence of constipation and feeding issues/food selectivity, but there was no association between autism case status and overall incidence of GI symptoms, diarrhoea, gastroesophageal reflux/vomiting, or abdominal. bloating/discomfort/irritability. | No adjustment for confounders. |

| **Supplementary table 2.** *List of confounders, variable type, descriptions, and methods of assessment* | | | |
| --- | --- | --- | --- |
| **Confounders** | **Variable type** | **Description** | **Method of assessment** |
| Parity | Categorical | 0*  1  2 or more | Questionnaire |
| Child’s sex | Binary | Female*  Male | Questionnaire |
| Family social class | Binary | Non-manual*  Manual | Questionnaire |
| Home ownership status | Binary | Mortgaged/owned*  Rented / other | Questionnaire |
| Mother highest educational attainment | Categorical | A-level or higher*  O-level  CSE/Vocational or less | Questionnaire |
| Maternal age at delivery | Continuous | 15-44 | Questionnaire |
| Child’s developmental level at 18 months | Continuous score | -7.49 to 2.86 | Developed from the ALSPAC questionnaire |
| Maternal antenatal depression score (measured at 18 weeks and 32 weeks of gestation) | Continuous score | 0-30 | ALSPAC questionnaire using the Edinburgh Postnatal Depression Scale (EPDS) |
| Maternal postnatal depression score (measured at 8 weeks and 8 months after delivery) | Continuous score | 0-30 | ALSPAC questionnaire using the Edinburgh Postnatal Depression Scale (EPDS) |
| Maternal antenatal anxiety score (measured at 18 weeks of gestation) | Continuous score | 0-16 | ALSPAC questionnaire using the Crown-Crisp anxiety scale |
| Maternal postnatal anxiety score (measured at 8 weeks after delivery) | Continuous score | 0-16 | ALSPAC questionnaire using the Crown-Crisp anxiety scale |
| Financial difficulty score | Continuous score | 0-15 | ALSPAC questionnaire |
| **Notes**:  ALSPAC-Avon Longitudinal Study of Parents and Children  *Reference category | | |  |

| **Supplementary table 3**. *The distribution of confounders and missing data across the samples shown in Fig.1 for the age 9 years data* | | | | |
| --- | --- | --- | --- | --- |
| **Variable** | **Initial number of participants in the study (<15645)** | **Participants with data for at least one autistic trait (15138)** | **Participants with data for at least one autistic trait and outcome variables (6708)** | **Participants with data for at least one autistic trait and outcome variables plus confounders (4233)** |
| **Confounders** | **N (%)** | **N (%)** | **N (%)** | **N (%)** |
| **SES** | **N = 11551** | **N = 11102** | **N = 6014** | **N = 4233** |
| Ref cat=non-manual |  |  |  |  |
| Manual | 2245 (19.44) | 2162(19.47) | 819 (13.62) | 528 (12.48) |
| Missing | 4094 (26.17) * | 4036 (26.66) * | 694 (10.35) * |  |
| **Home ownership** | **N = 9679** | **N = 9212** | **N = 5927** | **N = 4233** |
| Ref cat=Mortgaged/owned |  |  |  |  |
| Rented/Other | 1968 (20.33) | 1869 (20.29) | 902 (15.22) | 552 (13.05) |
| Missing | 5966 (38.13) * | 5926 (39.15) * | 781 (11.64) * |  |
| **Maternal education** | **N = 12470** | **N = 11982** | **N = 6277** | **N = 4233** |
| Ref cat=A-level or greater |  |  |  |  |
| O-level | 4319 (34.63) | 4156(34.69) | 2231 (35.54) | 1539 (36.37) |
| Certificate of Secondary Education, Vocational or less | 3753 (30.10) | 3600 (30.05) | 1302 (20.74) | 791 (18.70) |
| Missing | 3175 (20.29) * | 3156 (20.85) * | 431 (6.43) * |  |
| **Child sex** | **N = 15030** | **N = 14524** | **N =6708** | **N = 4233** |
| Ref cat=Female |  |  |  |  |
| Male | 7684 (51.12) | 7334 (50.49) | 3329 (49.63) | 2160 (51.05) |
| Missing | 615 (3.93) * | 614 (4.06) * |  |  |
| **Parity** | **N = 13101** | **N = 12613** | **N = 6252** | **N = 4233** |
| Ref cat=0 |  |  |  |  |
| 1 | 4576 (34.93) | 4391 (34.81) | 2231 (35.68) | 1502 (35.50) |
| 2 or more | 2661 (20.31) | 2553 (20.24) | 1059 (16.94) | 676 (15.98) |
| Missing | 2544 (16.26) * | 2525 (16.68) * | 456 (6.80) * |  |
| **Notes**:  Ref cat-reference category  SES-Socioeconomic status  *The proportions of missing values were calculated from the total number of participants for each column. | | | | |

| **Supplementary table 4**. *The distribution of confounders and missing data across the samples shown in Fig.1 for the age 14 years data* | | | | |
| --- | --- | --- | --- | --- |
| **Variable** | **Initial number of participants in the study**  **(<15645)** | **Participants with data for at least one autistic trait (15138)** | **Participants with data for at least one autistic trait and outcome variables (5555)** | **Participants with data for at least one autistic trait and outcome variables plus confounders (3514)** |
| **Confounders** | **N (%)** | **N (%)** | **N (%)** | **N (%)** |
| **SES** | **N = 11551** | **N = 11102** | **N = 4993** | **N = 3514** |
| Ref cat=non-manual |  |  |  |  |
| Manual | 2245 (19.44) | 2162(19.47) | 629 (12.60) | 410 (11.67) |
| Missing | 4094 (26.17) * | 4036 (26.66) * | 562 (10.12) * |  |
| **Home ownership** | **N = 9679** | **N = 9212** | **N = 4902** | **N = 3514** |
| Ref cat=Mortgaged/owned |  |  |  |  |
| Rented/other | 1968 (20.33) | 1869 (20.29) | 704 (14.36) | 441 (12.55) |
| Missing | 5966 (38.13) * | 5926 (39.15) * | 653 (11.76) * |  |
| **Maternal education** | **N = 12470** | **N = 11982** | **N = 5189** | **N = 3514** |
| Ref cat=A-level or greater |  |  |  |  |
| O-level | 4319 (34.63) | 4156(34.69) | 1819 (35.05) | 1261 (35.89) |
| Certificate of Secondary Education, Vocational or less | 3753 (30.10) | 3600 (30.05) | 988 (19.04) | 595 (16.93) |
| Missing | 3175 (20.29) * | 3156 (20.85) * | 366 (6.59) * |  |
| **Child sex** | **N = 15030** | **N = 14524** | **N = 5555** | **N = 3514** |
| Ref cat=Female |  |  |  |  |
| Male | 7684 (51.12) | 7334 (50.49) | 2570 (46.26) | 1695 (48.24) |
| Missing | 615 (3.93) * | 614 (4.06) * |  |  |
| **Parity** | **N = 13101** | **N = 12613** | **N = 5178** | **N = 3514** |
| Ref cat=0 |  |  |  |  |
| 1 | 4576 (34.93) | 4391 (34.81) | 1814 (35.03) | 1237 (35.20) |
| 2 or more | 2661 (20.31) | 2553 (20.24) | 857 (16.55) | 549 (15.62) |
| Missing | 2544 (16.26) * | 2525 (16.68) * | 377 (6.79) * |  |
| **Notes**:  Ref cat-reference category  SES-Socioeconomic status  *The proportions of missing values were calculated from the total number of participants for each column. | | | | |

| **Supplementary table 5.** *The proportions of confounders, incontinence/constipation in children with each autistic trait/diagnosed autism for the age 9 years data* | | | | | | | | | | | | | | | | | | |
| --- | --- | --- | --- | --- | --- | --- | --- | --- | --- | --- | --- | --- | --- | --- | --- | --- | --- | --- |
| **Variable** | **Social communications** | | | **Coherence** | | | **Sociability** | | | **Repetitive behaviour** | | | **Diagnosed autism** | | | **Factor-mean-score** | | |
|  | **Non-cases**  **N (%)** | **Cases N (%)** | **Chi^2^**  **P-value** | **Non-cases**  **N (%)** | **cases**  **N (%)** | **Chi^2^**  **P-value** | **Non-cases**  **N (%)** | **Cases**  **N (%)** | **Chi^2^**  **P-value** | **Non-cases**  **N (%)** | **Cases**  **N (%)** | **Chi^2^**  **P-value** | **Non-cases**  **N (%)** | **Cases**  **N (%)** | **Chi^2^**  **P-value** | **Non-cases**  **N (%)** | **Cases**  **N (%)** | **Chi^2^**  **P-value** |
| **Confounders** | | | | | | | | | | | | | | | | | | |
| **Social class** |  |  |  |  |  |  |  |  |  |  |  |  |  |  |  |  |  |  |
| manual | 459  (11.97) | 63 (15.83) | 0.03 | 505 (12.44) | 62 (15.01) | 0.13 | 499 (12.76) | 55 (11.18) | 0.32 | 488 (12.34) | 31 (10.76) | 0.43 | 566 (12.77) | <5* | 0.09 | 510  (12.42) | 59  (15.32) | 0.10 |
| non-manual | 3,376 (88.03) | 335 (84.17) |  | 3,555 (87.56) | 351 (84.99) |  | 3,411 (87.24) | 437 (88.82) |  | 3,467 (87.66) | 257 (89.24) |  | 3,867 (87.23) | 54 |  | 3,595  (87.58) | 326  (84.68) |  |
| **Mum’s highest educational level** |  |  |  |  |  |  |  |  |  |  |  |  |  |  |  |  |  |  |
| Certificate of Secondary Education, vocational or less | 703 (18.33) | 98 (24.62) | 0.01 | 757 (18.65) | 104 (25.18) | 0.004 | 727 (18.59) | 118 (23.98) | 0.003 | 736 (18.61) | 56 (19.44) | 0.92 | 858 (19.35) | 6  (10.53) | 0.24 | 775  (18.88) | 89  (23.12) | 0.10 |
| O-level | 1,387 (36.17) | 133 (33.42) |  | 1,476 (36.35) | 147 (35.59) |  | 1,415 (36.19) | 185 (37.60) |  | 1,440 (36.41) | 102 (35.42) |  | 1,607 (36.25) | 23 (40.35) |  | 1,491  (36.32) | 139  (36.10) |  |
| A-level or greater | 1,745 (45.50) | 167 (41.96) |  | 1,827 (45.00) | 162 (39.23) |  | 1,768 (38.41) | 189 (45.22) |  | 1779  (44.98) | 130  (45.14) |  | 1,968 (44.39) | 28 (49.12) |  | 1,839  (44.80) | 157  (40.78) |  |
| **Home ownership** |  |  |  |  |  |  |  |  |  |  |  |  |  |  |  |  |  |  |
| rented/other | 464 (12.10) | 69 (17.34) | 0.003 | 521 (12.83) | 64 (15.50) | 0.13 | 506 (12.94) | 60 (12.20) | 0.641 | 484 (12.24) | 40 (13.89) | 0.41 | 588 (13.26) | <5* | 0.003 | 521  (12.69) | 67  (17.40) | 0.01 |
| mortgaged/owned | 3,371 (87.90) | 329 (82.66) |  | 3,539 (87.17) | 349 (84.50) |  | 3,404 (87.06) | 432 (87.80) |  | 3,471 (87.76) | 248 (86.11) |  | 3,845 (86.74) | 57 |  | 3,584  (87.31) | 318  (82.60) |  |
| **Parity** |  |  |  |  |  |  |  |  |  |  |  |  |  |  |  |  |  |  |
| 0 | 1,854 (48.34) | 186 (46.73) | 0.43 | 1,980 (48.77) | 171 (41.40) | 0.02 | 1,924 (49.21) | 194 (39.43) | <0.001 | 1,898 (47.99) | 139 (48.26) | 0.93 | 2,130 (48.05) | 27 (47.37) | 0.87 | 2,001  (48.75) | 156  (40.52) | 0.01 |
| 1 | 1,374 (35.83) | 139 (34.92) |  | 1,429 (35.20) | 170 (41.16) |  | 1,365 (34.91) | 208 (42.28) |  | 1,424 (36.01) | 101 (35.07) |  | 1,586 (35.78) | 22 (38.60) |  | 1,454  (35.42) | 154  (40.00) |  |
| 2 or more | 607 (15.83) | 73 (18.34) |  | 651 (16.03) | 72 (17.43) |  | 621 (15.88) | 90 (18.29) |  | 633 (16.01) | 48 (16.67) |  | 717 (16.17) | 8 (14.04) |  | 650  (15.83) | 75  (19.48) |  |
| **Child’s sex** |  |  |  |  |  |  |  |  |  |  |  |  |  |  |  |  |  |  |
| female | 1,895 (49.41) | 138 (34.67) | <0.001 | 1,994 (49.11) | 146 (35.35) | <0.001 | 1,913 (48.93) | 197 (40.04) | <0.001 | 1,935 (48.93) | 106 (36.81) | <0.001 | 2,135 (48.16) | 14 (24.56) | <0.001 | 2,049  (49.91) | 100  (25.97) | <0.001 |
| male | 1940 (50.59) | 260 (65.33) |  | 2,066 (50.89) | 267 (64.65) |  | 1,997 (51.07) | 295 (59.96) |  | 2,020 (51.07) | 182 (63.19) |  | 2,298 (51.84) | 43 (75.44) |  | 2,056  (50.09) | 285  (74.03) |  |
| **Incontinence/constipation** | | | | | | | | | | | | | | | | | | |
| **Bedwetting** |  |  |  |  |  |  |  |  |  |  |  |  |  |  |  |  |  |  |
| Cases | 359  (9.36) | 62 (15.58) | <0.001 | 379 (9.33) | 61 (14.77) | <0.001 | 378 (9.67) | 57 (11.59) | 0.18 | 378 (9.56) | 37 (12.85) | 0.07 | 430 (9.70) | 11 (19.30) | 0.02 | 379  (9.23) | 62  (16.10) | <0.001 |
| Non-cases | 3,476 (90.64) | 336 (84.42) |  | 3,681 (90.67) | 352 (85.23) |  | 3,532 (90.33) | 435 (88.41) |  | 3,577 (90.44) | 251 (87.15) |  | 4,003 (90.30) | 46 (80.70) |  | 3,726  (90.77) | 323  (83.90) |  |
| **Daytime wetting** |  |  |  |  |  |  |  |  |  |  |  |  |  |  |  |  |  |  |
| Cases | 161  (4.20) | 39  (9.80) | <0.001 | 174 (4.29) | 40  (9.69) | <0.001 | 185 (4.73) | 24  (4.88) | 0.89 | 175 (4.42) | 20  (6.94) | 0.05 | 207 (4.67) | 8  (14.04) | 0.001 | 183  (4.46) | 32  (8.31) | 0.001 |
| Non-cases | 3,674 (95.80) | 359 (90.20) |  | 3,886 (95.71) | 373 (90.31) |  | 3,725 (95.27) | 468 (95.12) |  | 3,780 (95.58) | 268 (93.06) |  | 4,226 (95.33) | 49 (85.96) |  | 3,922  (95.54) | 353  (91.69) |  |
| **Soiling** |  |  |  |  |  |  |  |  |  |  |  |  |  |  |  |  |  |  |
| Cases | 177  (4.62) | 45 (11.31) | <0.001 | 186 (4.58) | 47 (11.38) | <0.001 | 196 (5.01) | 33  (6.71) | 0.11 | 196 (4.96) | 22  (7.64) | 0.05 | 228 (5.14) | 8  (14.04) | 0.003 | 185  (4.51) | 51  (13.25) | <0.001 |
| Non-cases | 3,658 (95.38) | 353 (88.69) |  | 3,874 (95.42) | 366 (88.62) |  | 3,714 (94.99) | 459 (93.29) |  | 3,759 (95.04) | 266 (92.36) |  | 4,205 (94.86) | 49 (85.96) |  | 3,920  (95.49) | 334  (86.75) |  |
| **Constipation** |  |  |  |  |  |  |  |  |  |  |  |  |  |  |  |  |  |  |
| Cases | 358  (9.34) | 47 (11.81) | 0.11 | 379 (9.33) | 44 (10.65) | 0.38 | 375 (9.59) | 41  (8.33) | 0.37 | 375 (9.48) | 29 (10.07) | 0.74 | 420 (9.47) | 5  (8.77) | 0.86 | 373  (9.09) | 52  (13.51) | 0.01 |
| Non-cases | 3,477 (90.66) | 351 (88.19) |  | 3,681 (90.67) | 369 (89.35) |  | 3,535 (90.41) | 451 (91.67) |  | 3,580 (90.52) | 259 (89.93) |  | 4,013 (90.53) | 52 (91.23) |  | 3,732  (90.91) | 333  (86.49) |  |
| *Notes*: *cell size may include zero or less than five, hence we did not present the percentages.  For social communication and coherence, cases were those in the worst 10% of the SCDC score and the coherence subscale respectively. For sociability, cases were those in the worst 11% score of the sociability scale. Cases for repetitive behaviours were those in the worst 7% of the repetitive behaviour scale. Non-cases were individuals below the thresholds indicated for each trait. For diagnosed autism, cases were those who had a confirmed diagnosis of autism while non-cases were those who did not have a confirmed diagnosis. For the factor-mean-score, cases were those in the worst 10% score. | | | | | | | | | | | | | | | | | | |

| **Supplementary table 6***. The proportions of confounders, incontinence/constipation in children with each autistic trait/diagnosed autism for the age 14 years data* | | | | | | | | | | | | | | | | | | | |
| --- | --- | --- | --- | --- | --- | --- | --- | --- | --- | --- | --- | --- | --- | --- | --- | --- | --- | --- | --- |
| **Variable** | **Social-communication difficulties** | | | **Coherence difficulties** | | | **Sociability difficulties** | | | **Repetitive behaviour** | | | **Diagnosed autism** | | | **Factor-mean-scores** | | | |
|  | **Non-cases**  **N (%)** | **Cases N (%)** | **Chi^2^**  **P-value** | **Non-cases**  **N (%)** | **cases**  **N (%)** | **Chi^2^**  **P-value** | **Non-cases**  **N (%)** | **Cases**  **N (%)** | **Chi^2^**  **P-value** | **Non-cases**  **N (%)** | **Cases**  **N (%)** | **Chi^2^**  **P-value** | **Non-cases**  **N (%)** | **Cases**  **N (%)** | **Chi^2^**  **P-value** | **Non-cases**  **N (%)** | **Cases**  **N (%)** | | **Chi^2^**  **P-value** |
| **Confounders** | | | | | | | | | | | | | | | | | | | |
| **Social class** |  |  |  |  |  |  |  |  |  |  |  |  |  |  |  |  |  | |  |
| manual | 344  (10.84) | 39  (14.03) | 0.11 | 348  (11.01) | 38  (12.42) | 0.46 | 380  (11.74) | 37  (9.81) | 0.27 | 370  (11.32) | 24  (11.06) | 0.91 | 432  (11.79) | <5* | 0.13 | 384  (11.25) | 49  (17.25) | | 0.003 |
| non-manual | 2,829  (89.16) | 239  (85.97) |  | 2,812  (88.99) | 268  (87.58) |  | 2,857  (88.26) | 340  (90.19) |  | 2,899  (88.68) | 193  (88.94) |  | 3,233  (88.21) | 31 |  | 3,029  88.75 | 235  82.75 | |  |
| **Mum’s highest educational level** |  |  |  |  |  |  |  |  |  |  |  |  |  |  |  |  |  | |  |
| Certificate of Secondary Education, vocational or less | 512  (16.14) | 55  (19.78) | 0.20 | 512  (16.20) | 68  (22.22) | 0.03 | 543  (16.77) | 76  (20.16) | 0.14 | 544  (16.64) | 38  (17.51) | 0.93 | 630  (17.19) | 6  (18.75) | 0.24 | 570  (16.70) | 66  (23.24) | | 0.02 |
| O-level | 1,141  (35.96) | 89  (32.01) |  | 1,123  (35.54) | 104  (33.99) |  | 1,153  (35.62) | 139  (36.87) |  | 1,175  (35.94) | 76  (35.02) |  | 1,314  (35.85) | 7  (21.88) |  | 1,231  (36.07) | 90  (31.69) | |  |
| A-level or greater | 1,520  (47.90) | 134  (48.20) |  | 1,525  (48.26) | 134  (43.79) |  | 1,541  (47.61) | 162  (42.97) |  | 1,550  (47.42) | 103  (47.47) |  | 1,721  (46.96) | 19  (59.38) |  | 1,612  (47.23) | 128  (45.07) | |  |
| **Home status** |  |  |  |  |  |  |  |  |  |  |  |  |  |  |  |  |  | |  |
| rented/other | 353  (11.13) | 42  (15.11) | 0.05 | 361  (11.42) | 38  (12.42) | 0.60 | 399  (12.33) | 36  (9.55) | 0.12 | 379  (11.59) | 34  (15.67) | 0.07 | 459  (12.52) | <5* | 0.03 | 420  (12.31) | 39  (13.73) | | 0.48 |
| mortgaged/owned | 2,820  (88.87) | 236  (84.89) |  | 2,799  (88.58) | 268  (87.58) |  | 2,838  87.67 | 341  90.45 |  | 2,890  (88.41) | 183  (84.33) |  | 3,206  (87.48) | 32 |  | 2,993  (87.69) | | 245  (86.27) |  |
| **Parity** |  |  |  |  |  |  |  |  |  |  |  |  |  |  |  |  | |  |  |
| 0 | 1,562  (49.23) | 133  (47.84) | 0.83 | 1,563  (49.46) | 130  (42.48) | 0.04 | 1,620  (50.05) | 154  (40.85) | 0.002 | 1,592  (48.70) | 112  (51.61) | 0.60 | 1,788  (48.79) | 18 | 0.56 | 1,685  (49.37) | | 121  (42.61) | 0.07 |
| 1 | 1,126  (35.49) | 99  (35.61) |  | 1,102  (34.87) | 128  (41.83) |  | 1,114  (34.41) | 162  (42.97) |  | 1,169  (35.76) | 76  (35.02) |  | 1,304  (35.58) | 11 |  | 1,205  (35.31) | | 110  (38.73) |  |
| 2 or more | 485  (15.29) | 46  (16.55) |  | 495  (15.66) | 48  (15.69) |  | 503  (15.54) | 61  (16.18) |  | 508  (15.54) | 29  (13.36) |  | 573  (15.63) | <5* |  | 1,685  (49.37) | | 121  (42.61) |  |
| **Child’s sex** |  |  |  |  |  |  |  |  |  |  |  |  |  |  |  |  | |  |  |
| female | 1,648  (51.94) | 109  (39.21) | <0.001 | 1,648  (52.15) | 113  (36.93) | <0.001 | 1,668  (51.53) | 167  (44.30) | 0.01 | 1,686  51.58 | 87  40.09 | 0.001 | 1,870  (51.02) | 9  (28.13) | 0.01 | 1,803  (52.83) | | 76  (26.76) | <0.001 |
| male | 1,525  (48.06) | 169  (60.79) |  | 1,512  (47.85) | 193  (63.07) |  | 1,569  48.47 | 210  55.70 |  | 1,583  48.42 | 130  59.91 |  | 1,795  (48.98) | 23  (71.88) |  | 1,610  (47.17) | | 208  (73.24) |  |
| **Incontinence/constipation** | | | | | | | | | | | | | | | | | | | |
| **Bedwetting** |  |  |  |  |  |  |  |  |  |  |  |  |  |  |  |  | |  |  |
| Cases | 74  (2.33) | 8  (2.88) | 0.57 | 69  (2.18) | 11  (3.59) | 0.12 | 75  (2.32) | 9  (2.39) | 0.93 | 80  (2.45) | <5* | 0.15 | 85  (2.32) | <5* | 0.76 | 70  (2.05) | | 16  (5.63) | <0.001 |
| Non-cases | 3,099  (97.67) | 270  (97.12) |  | 3,091  (97.82) | 295  (96.41) |  | 3,162  (97.68) | 368  (97.61) |  | 3,189  (97.55) | 215 |  | 3,580  (97.68) | 31 |  | 3,343  (97.95) | | 268  (94.37) |  |
| **Daytime wetting** |  |  |  |  |  |  |  |  |  |  |  |  |  |  |  |  | |  |  |
| Cases | 88  (2.77) | 9  (3.24) | 0.65 | 89  (2.82) | 12  (3.92) | 0.27 | 93  (2.87) | 11  (2.92) | 0.96 | 94  (2.88) | 5  (2.30) | 0.62 | 106  (2.89) | <5* | 0.94 | 92  (2.70) | | 15  (5.28) | 0.01 |
| Non-cases | 3,085  (97.23) | 269  (96.76) |  | 3,071  (97.18) | 294  (96.08) |  | 3,144  (97.13) | 366  (97.08) |  | 3,175  (97.12) | 212  (97.70) |  | 3,559  (97.11) | 31 |  | 3,321  (97.30) | | 269  (94.72) |  |
| **Soiling** |  |  |  |  |  |  |  |  |  |  |  |  |  |  |  |  | |  |  |
| Cases | 137  (4.32) | 18  (6.47) | 0.10 | 133  (4.21) | 24  (7.84) | 0.004 | 137  (4.23) | 24  (6.37) | 0.06 | 145  (4.44) | 13  (5.99) | 0.29 | 161  (4.39) | 5  (15.63) | 0.02 | 138  (4.04) | | 28  (9.86) | <0.001 |
| Non-cases | 3,036  (95.68) | 260  (93.53) |  | 3,027  (95.79) | 282  (92.16) |  | 3,100  (95.77) | 353  (93.63) |  | 3,124  (95.56) | 204  (94.01) |  | 3,504  (95.61) | 27  (84.38) |  | 3,275  (95.96) | | 256  (90.14) |  |
| **Constipation** |  |  |  |  |  |  |  |  |  |  |  |  |  |  |  |  | |  |  |
| Cases | 216  (6.81) | 34  (12.23) | 0.001 | 217  (6.87) | 37  (12.09) | 0.001 | 227  (7.01) | 35  (9.28) | 0.11 | 227  (6.94) | 30  (13.82) | 0.001 | 265  (7.23) | 5  (15.63) | 0.07 | 236  (6.91) | | 34  (11.97) | 0.002 |
| Non-cases | 2,957  93.19 | 244  87.77 |  | 2,943  (93.13) | 269  (87.91) |  | 3,010  (92.99) | 342  (90.72) |  | 3,042  (93.06) | 187  (86.18) |  | 3,400  (92.77) | 27  (84.38) |  | 3,177  (93.09) | | 250  (88.03) |  |
| *Notes*: *cell size may include zero or less than five, hence we did not present the percentages.  For social communication and coherence, cases were those in the worst 10% of the SCDC score and the coherence subscale respectively. For sociability, cases were those in the worst 11% score of the sociability scale. Cases for repetitive behaviours were those in the worst 7% of the repetitive behaviour scale. Non-cases were individuals below the thresholds indicated for each trait. For diagnosed autism, cases were those who had a confirmed diagnosis of autism while non-cases were those who did not have a confirmed diagnosis. For the factor-mean-score, cases were those in the worst 10% score. | | | | | | | | | | | | | | | | | | | |
